# Development of a Triage Tool for Stratification and Referral of Women at Risk of Preeclampsia in Low‐Resource Antenatal Settings: A Prospective Cohort Study

**DOI:** 10.1002/hsr2.71916

**Published:** 2026-02-26

**Authors:** Bismark Opoku Mensah, Ernestina Obenewaa Anim, Abena Serwaa Adjei

**Affiliations:** ^1^ Department of Biological sciences University of Worcester Worcester UK; ^2^ Department of Nursing All Nations University Koforidua Ghana; ^3^ Department of Population and Reproductive Health KNUST School of Public Health Kumasi Ghana; ^4^ Department of Public Health Komfo Anokye Teaching Hospital Kumasi Ghana

**Keywords:** antenatal care, clinical triage tool, prediction model, preeclampsia, risk stratification

## Abstract

**Background and Aims:**

Preeclampsia remains a leading cause of maternal and perinatal morbidity and mortality globally, with high incidence and case fatality rates in low‐ and middle‐income countries (LMICs). Early identification of at‐risk women is critical, yet many predictive models require laboratory or imaging resources that are unavailable in resource‐limited settings. This study aimed to develop a points‐based clinical triage tool for preeclampsia risk stratification using routinely collected antenatal care (ANC) data.

**Methods:**

A prospective cohort study of 703 pregnant women attending ANC was conducted, with retrospective extraction of baseline clinical data. Maternal sociodemographic characteristics, obstetric history, blood pressure (BP) measurements, and proteinuria screening results were analysed. Multivariable logistic regression was used to identify independent predictors of preeclampsia, and model performance was evaluated using discrimination, calibration, and internal bootstrapping. A weighted scoring system was derived from regression coefficients, and receiver operating characteristic (ROC) analysis was used to determine optimal cut‐off values for risk stratification.

**Results:**

A five‐component clinical triage tool for risk stratification of preeclampsia was developed. The tool incorporates maternal age (≥ 35 years), nulliparity, elevated blood pressure (≥ 140/90 mmHg), family history of preeclampsia, and dipstick proteinuria (≥ + 1), generating a total risk score ranging from 0 to 18. Based on the risk score, women were categorised into low risk (0–10 points), moderate risk (11–15 points), and high risk (≥ 16 points) groups. The incidence of preeclampsia increased across these categories, from 5.1% in the low‐risk group to 30.2% among women classified as high risk. At the high‐risk threshold (≥ 16 points), the tool demonstrated good discriminatory performance (AUC = 0.83), with a sensitivity of 78.3% and a specificity of 83.3% for identifying women who subsequently developed preeclampsia.

**Conclusion:**

This study demonstrates that a simple, points‐based clinical triage tool using routinely collected antenatal data has potential for stratifying preeclampsia risk.

## 1 Introduction

Preeclampsia remains a major contributor to maternal and perinatal morbidity and mortality worldwide, especially in low‐ and middle‐income countries (LMICs) [1], where health system constraints complicate early detection and timely intervention [2]. Globally, it accounts for an estimated 76,000 maternal deaths and 500,000 perinatal deaths annually [3]. In Ghana, preeclampsia and its complications are among the leading causes of obstetric admissions and adverse maternal and perinatal outcomes [4, 5].

Despite extensive research into prediction models for preeclampsia, most studies have been conducted in high‐income countries, where diagnostic infrastructure and patient health‐seeking behaviours differ from those in LMICs. As a result, the prediction models emerging from these studies often rely on advanced biomarkers, placental imaging, or genetic profiling [6, 7] which have limited relevance in resource‐limited settings such as Ghana. Most of the frontline antenatal care in Ghana is delivered through Community‐based Health Planning and Services (CHPS) compounds, community clinics, and district hospitals [8, 9], where laboratory capacity is minimal, specialist supervision is rare, and providers rely heavily on basic clinical assessments [10, 11].

Although routine tools such as blood pressure measurement and dipstick proteinuria testing are widely utilised during antenatal care (ANC) visits [12], there is currently no context‐specific triage algorithm that combines these measurements with other established predictors of preeclampsia into a structured framework to guide referral decisions in Ghana. As a result, decision‐making at the primary care level often relies on individual clinical judgment, which may vary by experience, workload, and resource availability [13].

The development of a simple clinical risk score, derived from variables routinely collected at ANC visits is an underexplored but promising approach to supporting clinical decision‐making where access to laboratory or specialist services is limited.

This study therefore aims to address this gap by developing a simple risk stratification system to standardize the early identification and referral of high‐risk cases. The goal is to optimize the utility of routinely collected data to enhance clinical decision‐making among ANC providers operating at the lowest levels of the healthcare system.

## 2 Methods

### 2.1 Study Design and Setting

This study employed a prospective cohort design with retrospective baseline data extraction to develop a clinical triage tool for preeclampsia risk stratification. The study was conducted at selected Community‐based Health Planning and Services (CHPS) compounds, community health centers, and district hospitals in rural and peri‐urban districts of Ghana. These facilities were selected based on criteria such as antenatal patient volume and the availability of services such as blood pressure monitoring and dipstick urinalysis.

### 2.2 Study Population and Participant Enrolment

The study included pregnant women aged 15–45 years who presented for antenatal care (ANC) from August 2023 to December 2024. Eligibility required documented confirmation of gestational age by obstetric ultrasound performed at or before 16 weeks of gestation. For women attending ANC for the first time at enrolment who had not previously undergone ultrasound dating, a dating scan was performed at enrolment to confirm gestational age. To ensure complete follow‐up and outcome ascertainment, women who indicated plans to deliver outside the study facilities or catchment area, or who were temporary residents and unlikely to complete scheduled follow‐up visits, were not enrolled. Eligible women were consecutively recruited and followed prospectively throughout their pregnancy until delivery.

### 2.3 Inclusion Criteria

Pregnant women aged between 15 and 45 years were eligible for inclusion if they were attending antenatal care (ANC) at any of the selected study sites and had a singleton pregnancy. Eligibility required documented confirmation of gestational age by obstetric ultrasound performed at or before 16 weeks of gestation.

### 2.4 Exclusion Criteria

Women were excluded from the study if gestational age could not be confirmed by obstetric ultrasound performed at or before 16 weeks of gestation, either prior to or at the time of enrolment. Those who had a clinical diagnosis of preeclampsia or exhibited both hypertension (≥ 140/90 mmHg) and proteinuria (≥ + 1) at booking were excluded. Additional exclusion criteria included chronic hypertension, chronic renal disease, pre‐existing diabetes mellitus, other chronic systemic illnesses, multiple gestations, and enrolment in concurrent interventional research studies. Women who initiated ANC at the study facilities but indicated plans to deliver outside the study facilities, or those unable to attend scheduled follow‐up visits, were also excluded.

### 2.5 Ethical Considerations

The study was approved by the Committee on Human Research, Publications, and Ethics at Kwame Nkrumah University of Science and Technology (CHRPE/AP/677/23). Written informed consent was obtained from all participants prior to enrolment. For participants under 18 years of age, written informed assent was obtained from the participant, in addition to written informed consent from a parent or legal guardian. Confidentiality and data protection protocols were followed throughout the study. No procedures outside of routine ANC were introduced.

### 2.6 Sample Size Calculation

The sample size was determined using the events‐per‐variable (EPV) principle, which recommends a minimum of 10 outcome events per predictor variable in logistic regression modelling. The model was pre‐specified to include seven clinically relevant and routinely collected data such as maternal age, body mass index (BMI), parity, blood pressure, dipstick proteinuria, timing of first ANC visit, and family history of preeclampsia. These variables were selected based on their documented association with preeclampsia in prior studies [14, 15].

To satisfy the EPV criterion for seven predictors, a minimum of 70 outcome events was required. Based on an estimated prevalence of preeclampsia of 10% [16], the total sample size necessary to observe at least 70 cases was calculated as: *n* = *E*/*p* = 70/0.10 = 700; where *n* is the required sample size, *E* is the number of events (70), and *p* is the expected proportion of participants with the outcome (10%). To account for potential loss to follow‐up and incomplete records, a 5% inflation was applied, yielding a final target sample size of 735 participants.

### 2.7 Baseline Data Collection

At the point of enrolment, each participant underwent a structured clinical assessment conducted by trained antenatal care personnel. Baseline information was collected using a standardised case record form and included maternal age, parity, family history of preeclampsia and obstetric history. All core clinical variables were routinely documented in ANC records, while information on family history of preeclampsia and prior obstetric history were obtained prospectively at enrolment. Family history of preeclampsia was defined as self‐reported history of preeclampsia in a first‐degree female relative.

The weight and height were measured using the Garmin Index S2 Smart Scale and Seca 213 stadiometer respectively. Blood pressure was measured with the participant seated, using the Sejoy DBP‐6673B digital sphygmomanometer in accordance with standard ANC protocols. Urine dipstick proteinuria was assessed during routine urinalysis and categorised as negative, trace, +1, or higher. The timing of the first ANC visit was recorded and classified as early (< 12 weeks of gestation) or late (≥ 12 weeks).

Gestational age was confirmed for all participants using first or early second‐trimester obstetric ultrasound scans. For women who had previously attended ANC, ultrasound dating results were extracted from the ANC records. For participants who were presenting for their first ANC visit at the time of enrolment and had not yet undergone an ultrasound scan, a dating scan was performed at enrolment using the SonoScape S11 Plus ultrasound system (SonoScape Medical Corp., Shenzhen, China) by a certified sonographer.

All collected data were double‐entered into a secure, password‐protected electronic database. Cross‐verification procedures were applied to ensure internal consistency, minimise transcription errors, and maintain data integrity prior to analysis.

### 2.8 Follow‐up and Outcome Ascertainment

Participants were followed throughout subsequent ANC visits until delivery. Outcome ascertainment was performed through review of ANC records, facility delivery registers, and postnatal discharge summaries. The primary outcome was preeclampsia, defined in accordance with WHO and Ghana Health Service criteria as new‐onset hypertension (≥ 140/90 mmHg) occurring after 20 weeks of gestation, accompanied by either proteinuria or features of maternal organ dysfunction. Women diagnosed with preeclampsia were recorded as outcome‐positive and all others were classified as outcome‐negative.

### 2.9 Development of a Scoring System

Regression coefficients from multivariable logistic regression model were transformed into an integer‐based scoring system. Each *β*‐coefficient was divided by a constant of 0.5 [17, 18] and then rounded to the nearest whole number.

### 2.10 Statistical Analysis

All analyses were conducted using SPSS version 19. Descriptive statistics were used to summarise demographic and clinical characteristics data. Predictor variables were pre‐specified based on clinical relevance and availability in routine antenatal care. These variables were entered into multivariable logistic regression models, and statistical significance (*p* < 0.05) was used to identify independent predictors retained in the final model.

A points‐based risk score was derived from the final regression model. Risk score categories were defined based on the distribution of total scores, observed incidence of preeclampsia across score ranges, and receiver operating characteristic (ROC) curve analysis, with the optimal ROC‐derived cut‐off used to define the high‐risk group. The model's performance was assessed using ROC curve analysis while calibration was evaluated using calibration plot and the Hosmer–Lemeshow goodness‐of‐fit test.

## 3 Results

### 3.1 Baseline Characteristics of the Study Population

Of the 735 women enrolled, 703 (95.6%) had complete baseline data for all candidate predictors and complete outcome ascertainment and were included in the final analysis. The remaining 32 (4.4%) participants were excluded due to incomplete follow‐up or unavailable delivery outcome data. Reasons for loss to follow‐up included relocation outside the study catchment area, transfer of care to non‐participating facilities, and inability to attend scheduled antenatal visits.

Among the 703 women included in the analysis, 96 (13.7%) developed preeclampsia (PE), 59 (8.4%) developed gestational hypertension (GH), and 548 (78.0%) remained normotensive throughout pregnancy.

The mean maternal age across the cohort was 30.88 (± 6.13) years, with no significant differences observed among the preeclamptic and normotensive groups (*p* = 0.90). However, a significantly higher proportion of women with preeclampsia (51.0%) were of advanced maternal age (≥ 35 years), compared to 39.2% among normotensives (*p* = 0.01). Parity did not differ significantly between the groups (*p* = 0.50), although a lower proportion of nulliparous women was observed among those with preeclampsia (35.4%) compared to normotensive participants (55.1%).

Body mass index (BMI) at booking showed no statistically significant variation across groups (*p* = 0.29). Additionally, the proportion of participants with BMI ≥ 30 kg/m² was comparable among preeclampsia (45.8%) and normotensive groups (42.0%) (*p* = 0.78).

Although women diagnosed with preeclampsia had a mean booking systolic blood pressure within normotensive thresholds (125.17 ± 6.54 mmHg), it was significantly higher compared to normotensive participants (117.21 ± 6.01 mmHg, *p* < 0.001). No significant difference was observed in mean diastolic blood pressure across the preeclamptic and normotensive groups (*p* = 0.10).

Late antenatal care (ANC) initiation was common across the cohort (56.5%) but significantly more frequent among preeclamptic women (63.5%) than normotensive women (55.1%) (*p* < 0.001). The proportion of participants reporting a family history of preeclampsia was similar across all groups. However, prior adverse obstetric history was significantly more common (*p *= 0.02) among preeclamptic women (19.8%) compared to normotensive women (6.2%) and was absent in the gestational hypertension group (Table 1).

**Table 1:** Baseline sociodemographic and clinical characteristics of study participants.

Variable	Total (*n* = 703)	PE (*n* = 96)	GH (*n* = 59)	Normotensive (*n* = 548)	*p* value*
Maternal age (years), mean (SD)	30.88 ± 6.13	30.42 ± 6.42	30.20 ± 5.64	29.96 ± 6.27	0.90
≥ 35 years *n* (%)	294 (41.8%)	49 (51.0%)	30 (50.8%)	215 (39.2%)	**0.01**
Nulliparity *n* (%)	360 (51.2%)	34 (35.4%)	24 (40.7%)	302 (55.1%)	0.50
BMI at booking (kg/m^2^), Median (IQR)	30.6 (24.35–39.52)	29.76 (24.25–40.75)	29.33 (25.45–38.28)	30.28 (23.07–38.66)	0.29
BMI ≥ 30 kg/m² *n* (%)	302 (43.0%)	44 (45.8%)	28 (47.5%)	230 (42.0%)	0.78
Booking systolic BP (mmHg), mean (SD)	127.21 ± 7.08	125.17 ± 6.54	127.09 ± 7.00	117.21 ± 6.01	**< 0.001**
Booking diastolic BP (mmHg), mean (SD)	83.44 ± 5.95	83.15 ± 5.26	82.94 ± 5.91	82.76 ± 5.58	0.10
Late ANC initiation (> 12 weeks) *n* (%)	397 (56.5%)	61 (63.5%)	34 (57.6%)	302 (55.1%)	**< 0.001**
Family history of PE *n* (%)	263 (37.4%)	36 (37.5%)	23 (39.0%)	204 (37.2%)	0.89
Prior adverse obstetric history *n* (%)	53 (7.5%)	19 (19.8%)	0 (0.0%)	34 (6.2%)	**0.02**
**Marital status *n* (%)**					0.78
Married	442 (62.9%)	57 (59.4%)	37 (62.7%)	348 (63.5%)	
Single	201 (28.6%)	22 (22.9%)	14 (23.7%)	165 (30.1%)	
Co‐habiting	60(8.5%)	17 (17.7%)	8 (13.6%)	35 (6.4%)	
**Educational level *n* (%)**					0.89
No formal education	34 (4.8%)	9 (9.4%)	1 (1.7%)	24 (4.4%)	
Basic education	77 (11.0%)	19 (19.8%)	21 (35.6%)	37 (6.8%)	
Secondary	475 (67.6%)	48 (50.0%)	27 (45.8%)	400 (72.9%)	
Tertiary	117 (16.6%)	20 (20.8%)	10 (16.9%)	87 (15.9%)	
**Employment status *n* (%)**					0.41
Unemployed	48 (6.8%)	5 (5.2%)	11 (18.6%)	32 (5.8%)	
Informal sector	460 (65.4%)	51 (53.1%)	32 (54.2%)	377 (68.8%)	
Formal sector	195 (27.7%)	40 (41.7%)	16 (27.1%)	139 (25.4%)	
**Residence**					0.25
Urban	174 (24.8%)	37 (38.5%)	29 (49.2%)	108 (19.7%)	
Peri‐urban	237 (33.7%)	29 (30.2%)	11 (18.6%)	197 (36.0%)	
Rural	292 (41.5%)	30 (31.3%)	19 (32.2%)	243 (44.3%)	

Abbreviations: ANC, antenatal care; GE, gestational hypertension; IQR, interquartile range; PE, preeclampsia; SD, standard deviation.

* *p*‐values for comparisons between PE and normotensive groups, Boldened *p*‐values are statistically significant.

### 3.2 Blood Pressure Trends Across Gestation and Postpartum

The mean systolic blood pressure (SBP) and diastolic blood pressure (DBP) were analysed across five key timepoints; early pregnancy (1–13 weeks), mid‐pregnancy (14–27 weeks), late pregnancy (28–40 weeks), at delivery, and postpartum (2–3 days).

SBP levels increased progressively across gestation in the preeclamptic and gestational hypertension groups, with the most pronounced increase observed among women with preeclampsia. The preeclampsia group exhibited a marked elevation in mean SBP from 123 mmHg at 1–13 weeks to a peak of 161 mmHg at delivery, followed by a decline to 140 mmHg postpartum. Gestational hypertension showed a more gradual rise in SBP, reaching 139 mmHg at delivery and falling to 124 mmHg postpartum. In contrast, the normotensive group maintained relatively stable SBP across all timepoints, ranging from 116 to 122 mmHg.

**Figure 1:**
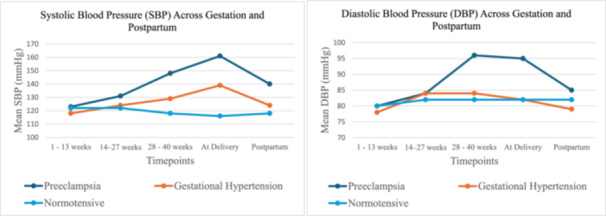
Trends in blood pressure at different timepoints.

DBP patterns followed a similar trajectory. Women with preeclampsia recorded substantial elevations in DBP during late pregnancy, peaking at 96 mmHg at 28–40 weeks and a steady decline postpartum. In contrast, gestational hypertension exhibited more moderate DBP increases, peaking at 84 mmHg and declining to 79 mmHg postpartum. The normotensive group maintained the lowest and most stable DBP profile, with values consistently between 80 and 82 mmHg (Figure 1).

### 3.3 Maternal Risk Factors Associated With Preeclampsia and Gestational Hypertension

After adjusting for covariates, several maternal risk factors were found to be independently associated with preeclampsia and gestational hypertension, although the strength and direction of these associations varied across the two conditions.

Advanced maternal age (≥ 35 years) was significantly associated with both preeclampsia and gestational hypertension. Women aged 35 years or older had 2.46 times higher odds of developing preeclampsia (95% CI: 1.28–3.14, *p* = 0.03), and 1.67 times higher odds of gestational hypertension (95% CI: 1.14–3.77, *p* = 0.004).

Obesity (BMI ≥ 30 kg/m²) was significantly associated with gestational hypertension (aOR = 1.95, 95% CI: 1.29–2.81, *p* = 0.01) but not with preeclampsia (aOR = 1.06, 95% CI: 0.57–1.96, *p* = 0.08). Similarly, late initiation of antenatal care (ANC) significantly increased the odds of gestational hypertension (aOR = 1.80, 95% CI: 1.02–2.89, *p* = 0.01) but not preeclampsia (aOR = 1.92, 95% CI: 0.92–3.04, *p* = 0.09).

Nulliparity was independently associated with increased risk of both preeclampsia (aOR = 2.10, 95% CI: 1.22–3.42, *p* = 0.005), and gestational hypertension (aOR = 1.40, 95% CI: 1.21–2.84, *p* = 0.02). A family history of preeclampsia was also associated with both conditions, with women reporting such a history having 2.01 times higher odds of developing preeclampsia (95% CI: 1.09–3.12, *p* = 0.03) and 3.11 times higher odds of developing gestational hypertension (95% CI: 1.02–4.04, *p* < 0.001).

In contrast, the number of ANC visits was not significantly associated with either condition (PE: aOR = 0.59, 95% CI: 0.15–2.30, *p* = 0.21; GH: aOR = 1.50, 95% CI: 0.30–2.46, *p* = 0.19) (Table 2).

**Table 2:** Multivariable analysis of maternal risk factors for preeclampsia and gestational hypertension.

Variable	PE vs normotensive aOR (95% CI)	*p*‐value	GH vs normotensive aOR (95% CI)	*p* value
Age ≥ 35 (years)	2.46 (1.28–3.14)	**0.030**	1.67 (1.14–3.77)	**0.004**
BMI ≥ 30 (kg/m^2^)	1.06 (0.57–1.96)	0.081	1.95 (1.29–2.81)	**0.01**
Elevated BP ≥ 140/90 (mmHg)	3.25 (1.19–5.58)	**< 0.001**	4.08 (2.11–6.18)	**< 0.001**
Dipstick proteinuria ≥ + 1	1.27 (1. 15–3.02)	**0.012**	0.67 (0.30–1.16)	0.21
Nulliparity	2.10 (1.22–3.42)	**0.005**	1.40 (1.21–2.84)	**0.02**
Late ANC booking (> 12 weeks)	1.92 (0.92–3.04)	0.09	1.80 (1.02–2.89)	**0.01**
Number of ANC visits	0.59 (0.15–2.30)	0.211	1.50 (0.30–2.46)	0.19
Family history of PE	2.01 (1.09–3.12)	**0.03**	3.11 (1.02–4.04)	**< 0.001**

*Note:* Boldened *p*‐values are statistically significant.

Abbreviations: ANC, antenatal care; aORs, adjusted odds ratios; CH, chronic hypertension; GE, gestational hypertension; PE, preeclampsia.

### 3.4 Performance of Dipstick Proteinuria and Elevated Blood Pressure in Screening for Preeclampsia

The performance of dipstick proteinuria (≥ + 1), elevated blood pressure (≥ 140/90 mmHg), and their combination was evaluated against the reference standard of clinical diagnosis of preeclampsia. When analysed separately, dipstick proteinuria showed modest clinical utility, with limited specificity (44.8%) and moderate sensitivity (66.1%). Elevated blood pressure however demonstrated a more balanced diagnostic profile, achieving both sensitivity and specificity estimates in the mid‐60% range (60.0% and 64.3%, respectively).

When elevated blood pressure and dipstick proteinuria were combined, the highest sensitivity of 75.0% and a negative predictive value of 85.5% were achieved. However, the specificity (63.2%) and positive predictive value (46.6%) did not significantly improve compared to dipstick proteinuria and blood pressure measurements alone. The combination approach resulted in the highest diagnostic accuracy at 66.8%, but the performance remained suboptimal (Table 3).

**Table 3:** Comparative screening performance of dipstick proteinuria, elevated blood pressure, and their combination in identifying preeclampsia.

Diagnostic metric	Dipstick ≥ + 1	BP ≥ 140/90 (mmHg)	BP + dipstick	*p* value
Sensitivity (%)	66.1 (53.7–76.7)	60.0 (51.1–68.3)	75.0 (66.6–81.9)	**0.04**
Specificity (%)	44.8 (32.7–57.5)	64.3 (58.5–69.7)	63.2 (57.4–68.6)	0.19
PPV (%)	56.2 (44.8–67.0)	41.9 (34.7–49.3)	46.6 (39.7–53.7)	0.11
NPV (%)	55.3 (41.2–68.6)	78.9 (73.2–83.7)	85.5 (80.1–89.7)	**< 0.001**
Accuracy (%)	55.8 (46.9–64.4)	63.0 (58.2–67.6)	66.8 (62.0–71.2)	**0.03**

*Note:* Values are shown with 95% Confidence Intervals.

Abbreviations: NPV, negative predictive value; PPV, positive predictive value.

### 3.5 Clinical Triage Model for Preeclampsia Risk Stratification

A multivariable logistic regression model was developed to identify key clinical predictors of preeclampsia and to construct a triage tool for use in resource‐limited antenatal care settings. The model included five binary predictors: maternal age ≥ 35 years, nulliparity, elevated blood pressure (≥ 140/90 mmHg), family history of preeclampsia, and dipstick proteinuria (≥ + 1).

All five predictors were independently associated with significantly increased odds of preeclampsia. Family history of preeclampsia and nulliparity showed strong associations with odds ratios (OR) of 8.33 and = 9.21, respectively. This was closely followed by advanced maternal age (OR = 8.31) and elevated blood pressure (OR = 6.69). Although dipstick proteinuria was statistically significant (*p* = 0.002), its effect size was comparatively modest (OR = 3.26) (Table 4).

**Table 4:** Logistic regression model for preeclampsia risk stratification.

Predictor	*β*	OR	95% CI	*p* value
Age ≥ 35 years	2.12	8.31	4.27–16.16	**< 0.001**
Nulliparity	2.22	9.21	6.32–13.42	**< 0.001**
Elevated BP ≥ 140/90 (mmHg)	1.90	6.69	2.37–18.90	**< 0.001**
Family history of PE	2.12	8.33	6.22–11.15	**< 0.001**
Dipstick proteinuria ≥ + 1	1.18	3.26	2.05–5.20	**0.002**

*Note:* Boldened *p*‐values are statistically significant.

Abbreviations: BP, blood pressure; PE, preeclampsia.

The model demonstrated good overall performance, with a Nagelkerke R² value of 0.783. The calibration result assessed by the Hosmer–Lemeshow test was satisfactory (*p* = 0.634) and the model achieved an overall classification accuracy of 89.3% with a sensitivity of 85.8% and a specificity of 81.8%.

**Figure 2:**
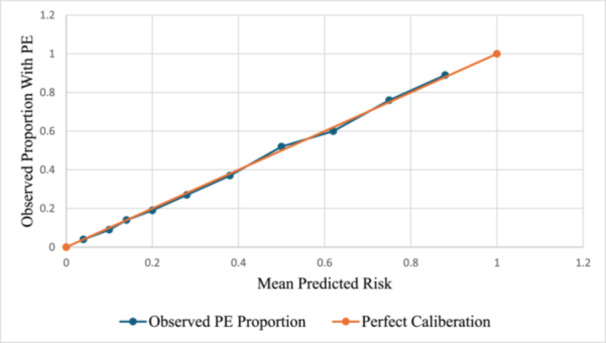
Calibration plot of the model for preeclampsia risk stratification.

Internal model validation was performed using non‐parametric bootstrapping. All predictors retained statistical significance (*p *< 0.05), and the bias‐corrected 95% confidence intervals supported the stability of the model coefficients (Table 5). The calibration of the model was further evaluated using a calibration plot, which compared the predicted probabilities of preeclampsia with the observed event rates across deciles of predicted risk. The resulting calibration curve closely followed the reference line (Figure 2).

**Table 5:** Bootstrap validation of logistic regression coefficients.

Predictor	Bootstrapped *β*	Bias	Std error	95% CI	*p* value
Age ≥ 35 years	2.08	0.15	0.54	2.80–23.10	**< 0.001**
Nulliparity	2.16	0.14	0.49	3.30–22.60	**< 0.001**
Elevated BP ≥ 140/90 (mmHg)	1.84	0.11	0.57	2.10–19.30	**0.002**
Family history of PE	2.89	0.16	0.55	6.10–23.20	**0.008**
Dipstick proteinuria ≥ + 1	1.20	0.22	0.09	1.01–1.42	**0.02**

*Note:* Boldened *p*‐values are statistically significant.

Abbreviations: BP, blood pressure; PE, preeclampsia.

A points‐based scoring system was derived from the regression model with each predictor assigned an integer score proportional to its regression coefficient. Family history of preeclampsia, advanced maternal age (≥ 35 years), nulliparity, and elevated blood pressure were each assigned 4 points. Dipstick proteinuria was assigned 2 points, resulting in a total score range of 0 to 18 (Table 6).

**Table 6:** Preeclampsia triage scoring system.

Predictor	*β*	Points
Age ≥ 35 years	2.12	4
Nulliparity	2.22	4
Elevated BP ≥ 140/90 (mmHg)	1.90	4
Family history of PE	2.12	4
Dipstick proteinuria ≥+1	1.18	2
Total possible score		18

ROC curve analysis determined an optimal cut‐off score of ≥ 16 for the model, which yielded a sensitivity of 78.3% and a specificity of 83.3% with an AUC of 0.83 for predicting preeclampsia (Table 7). The incidence of preeclampsia increased progressively across score ranges, from 5.1% among women scoring ≤ 5% to 30.2% among those scoring ≥ 16. Based on these distributions, three risk categories were defined as low risk (0–10 points), moderate risk (11–15 points), and high risk (≥ 16 points) (Table 8).

**Table 7:** Performance of triage model for predicting preeclampsia.

Metric	Value	95% CI
Cut‐off score	≥ 16	
Sensitivity (%)	78.3	64.4–87.7%
Specificity (%)	83.3	79.1–86.8%
PPV (%)	37.5	28.5–47.5%
NPV (%)	96.8	94.2–98.2%
AUC	0.83	0.78–0.89

Abbreviations: AUC, area under curve; NPV, negative predictive value; PPV, positive predictive value.

**Table 8:** Observed incidence of preeclampsia by score categories.

Score range	*N* (Participants)	PE cases (*n*)	Incidence of PE (%)
≤ 5	195	10	5.1%
6–10	156	5	5.1%
11–15	170	26	15.3%
≥ 16	182	55	30.2%

## 4 Discussion

This study aimed to develop a clinical triage tool for stratifying preeclampsia risk among pregnant women attending routine antenatal care (ANC). In a cohort of 703 pregnant women, the incidence of preeclampsia was 13.7%. This is higher than the global estimate of 2%–8% [19] and exceeds the reported national prevalence in Ghana of 6.6%–8.8% [20] However, this incidence falls within the sub‐Saharan Africa range, where the World Health Organization (WHO) estimates between 1.8% and 16.7% [21]. This finding highlights the pressing need for facility‐level, low‐cost risk stratification strategies that can be implemented effectively across the various regions in Ghana.

A significantly higher proportion of women with preeclampsia were of advanced maternal age (≥ 35 years) compared to their normotensive counterparts. This is consistent with findings from meta‐analyses which show that advanced maternal age increases the risk of preeclampsia [22, 23, 24]. The underlying mechanisms are thought to involve age‐related vascular and metabolic changes, increased prevalence of chronic comorbidities, and cumulative endothelial dysfunction [25].

Obesity (BMI ≥ 30 kg/m²) was associated with gestational hypertension but not with preeclampsia in this study. This finding contrasts with previous studies that report a strong association between obesity and both conditions [26, 27, 28]. This variation may be attributed to the timing of BMI assessment. In this cohort, variables for BMI assessment were recorded at the time of ANC initiation, which for many participants occurred after 12 weeks of gestation. As a result, the recorded BMI likely reflected gestational weight gain rather than pre‐pregnancy obesity, potentially resulting in misclassification of exposure status. This may have attenuated the association with preeclampsia.

Nulliparity was independently associated with increased odds of both preeclampsia and gestational hypertension. This aligns with longstanding evidence that first pregnancies have a higher risk for hypertensive disorders of pregnancy [29, 30]. Both immunological mechanisms in first pregnancies [31] and socio‐demographic correlates of nulliparity may account for this observation [32].

Late ANC initiation (≥ 12 weeks gestation) was common in this study (56.5%) and was prevalent among women with preeclampsia (63.5%). This pattern is consistent with national data on ANC attendance in Ghana [33] and reflects a combination of structural and socio‐economic barriers like long travel distances, transportation challenges, service costs, and sociocultural influences that delay early engagement with antenatal services [34, 35]. Addressing these barriers will enable earlier identification and timely intervention for women at risk of preeclampsia.

A family history of preeclampsia was independent predictor for preeclampsia and gestational hypertension, with higher odds ratios for gestational hypertension (aOR 3.11) than for preeclampsia (aOR 2.01). This finding is supported by genetic and familial aggregation studies reporting shared heritable risk for preeclampsia and gestational hypertension [36, 37]. This suggests that incorporating a structured family history checklist into ANC booking visits could enhance early risk flagging in settings where laboratory markers are inaccessible.

In this study, urine dipstick proteinuria at a threshold of ≥ 1+ demonstrated modest screening performance for preeclampsia, with moderate sensitivity (66.1%) but limited specificity (44.8%). These findings are consistent with reports from sub‐Saharan Africa where urine dipstick testing is widely used as a surrogate for laboratory‐based protein quantification [38, 39].

Elevated blood pressure (≥ 140/90 mmHg) demonstrated a more balanced diagnostic profile, aligning with the central role of hypertension in the diagnostic criteria for preeclampsia, as reported in the National Institute for Health and Care Excellence (NICE 2023) [40], International Society for the Study of Hypertension in Pregnancy (ISSHP 2021) [41], and American College of Obstetricians and Gynecologists (ACOG 2020) [42] guidelines. However, the moderate sensitivity indicates that a significant proportion of women with preeclampsia could not be detected. This limitation reinforces the importance of repeated BP assessments over time, a point further supported by the progressive blood pressure elevations observed in the trend analysis from this study.

When elevated blood pressure (≥ 140/90 mmHg) and dipstick proteinuria (≥ 1 + ) were combined, sensitivity improved to 75.0%, with the highest negative predictive value (85.5%) among the tested approaches, suggesting utility as a triage tool for preeclampsia screening in low‐resource settings. These results support the integration of combined blood pressure and proteinuria assessment within ANC workflows as an initial screening step for preeclampsia.

The multivariable logistic regression model developed in this study incorporated risk predictors such as advanced maternal age (≥ 35 years), nulliparity, elevated blood pressure (≥ 140/90 mmHg), family history of preeclampsia, and dipstick proteinuria (≥ 1+), all of which were independently associated with increased odds of preeclampsia.

The model's performance metrics indicate good predictive capacity, with an overall classification accuracy of 89.3%, sensitivity of 85.8%, and specificity of 81.8%. These results compare favourably with data from previously published clinical prediction models for preeclampsia based solely on clinical variables [43, 44, 45]. The model also demonstrated good calibration (Hosmer–Lemeshow *p* = 0.634) and stability of coefficients on internal validation, supporting its robustness for application in similar populations.

From a clinical implementation perspective, the strength of this model lies in its reliance on variables that are routinely collected in standard ANC visits, without the need for laboratory assays or specialised imaging. This feature enhances its feasibility in low‐resource settings, where predictive models incorporating biochemical markers, though often more accurate [46] are rarely scalable. However, while the predictive performance is encouraging, external validation in the larger Ghanaian population is necessary to ensure generalisability, given potential regional differences in risk factor prevalence, measurement quality, and disease patterns.

The operationalisation of this model could allow healthcare workers at all facility levels to stratify women into high, intermediate, and low‐risk groups for targeted surveillance and timely intervention. Integrating this tool into ANC workflows has the potential to improve early identification of at‐risk women while minimising unnecessary referrals in already overburdened healthcare systems.

While the findings of this study are promising, they should be interpreted considering certain limitations. The study was conducted within selected health facilities in a single region of Ghana. The model may therefore not fully capture variations in sociodemographic composition observed in other regions. A broader multi‐site validation and implementation studies are needed to assess its utility in routine antenatal care.

## 5 Conclusion

This study demonstrates that routine antenatal care data, when applied systematically, can enhance risk stratification for preeclampsia. By integrating routinely collected clinical data into a points‐based scoring system, this study has developed a simple triage tool that can be readily implemented within existing antenatal care workflows without additional infrastructure.

For the local population, this work provides the first context‐specific model for preeclampsia risk stratification that reflects the realities of antenatal service delivery in Ghana. The model's accuracy and operational simplicity offer a pathway to more targeted surveillance and prioritised referral of high‐risk women.

## Author Contributions

**Bismark Opoku Mensah:** conceptualization, formal analysis, methodology, resources, validation, writing – original draft. **Ernestina Obenewaa Anim:** investigation, methodology, writing – review and editing. **Abena Serwaa Adjei:** investigation, methodology, resources, writing – review and editing.

## Funding

The authors received no specific funding for this work.

## Conflicts of Interest

The authors declare no conflicts of interest.

## Transparency Statement

The lead author Bismark Opoku Mensah affirms that this manuscript is an honest, accurate, and transparent account of the study being reported; that no important aspects of the study have been omitted; and that any discrepancies from the study as planned (and, if relevant, registered) have been explained.

## Data Availability

The data that support the findings of this study are available from the corresponding author upon reasonable request.
